# Additively-Manufactured Broadband Metamaterial-Based Luneburg Lens for Flexible Beam Scanning

**DOI:** 10.3390/ma17122847

**Published:** 2024-06-11

**Authors:** Xuanjing Li, Rui Feng, Quilin Tan, Jianjia Yi, Shixiong Wang, Feng He, Shah Nawaz Burokur

**Affiliations:** 1 Key Laboratory of Micro/Nano Devices and Systems, Ministry of Education, North University of China, Taiyuan 030051, China; sz202106013@st.nuc.edu.cn (X.L.); ruifeng@nuc.edu.cn (R.F.); 2 The State Key Laboratory of Dynamic Measurement Technology, North University of China, Taiyuan 030051, China; 3 School of Electronics and Information Engineering, Xi’an Jiaotong University, Xi’an 710049, China; jianjia.yi@xjtu.edu.cn (J.Y.); sxwang@stu.xjtu.edu.cn (S.W.); 4 The 48th Research Institute of China Electronics Technology Group Corporation, Changsha 410111, China; hefeng@cs48.com; 5 LEME, Univ. Paris Nanterre, F92410 Ville d’Avray, France

**Keywords:** Luneburg lens, metamaterial, flexible antenna, 3D printing, multi-beam generation

## Abstract

Multi-beam microwave antennas have attracted enormous attention owing to their wide range of applications in communication systems. Here, we propose a broadband metamaterial-based multi-beam Luneburg lens-antenna with low polarization sensitivity. The lens is constructed from additively manufactured spherical layers, where the effective permittivity of the constituting elements is obtained by adjusting the ratio of dielectric material to air. Flexible microstrip patch antennas operating at different frequencies are used as primary feeds illuminating the lens to validate the radiation features of the lens-antenna system. The proposed Luneburg lens-antenna achieves ±72° beam scanning angle over a broad frequency range spanning from 2 GHz to 8 GHz and presents a gain between 15.3 dBi and 22 dBi, suggesting potential applications in microwave- and millimeter-wave mobile communications, radar detection and remote sensing.

## 1. Introduction

With the advance in wireless communication technologies, there is an increasing demand for high-data-rate transmissions. Owing to limited spectrum resources, attention has migrated towards multi-beam antennas capable of generating independent high-gain directional beams. Various methods to generate the multi-beams, such as reflectors [1,2,3] and phased-array antennas [4,5] have been proposed. In comparison, the Luneburg lens, which is a spherical gradient index lens capable of generating multiple independent beams, presents the advantages of low loss and broadband performances. Based on the Luneburg principle [6,7,8,9], the electromagnetic energy emitted by a feed source positioned on the lens periphery is radiated as a plane wave at the diagonally opposite side of the sphere. Similarly, an incident plane wave that passes through a lens will converge at the diagonally opposite point on the surface of the lens. Numerous techniques exist for beam focusing, including the utilization of surface plasmon polaritons in quasi-planar structures [10,11,12] and broadband achromatic lenses based on gradient metasurfaces [13,14,15].

The Luneburg lens has found extensive applications in radar, electronic warfare, wireless and satellite communication systems owing to its low cost and simple feeding networks [16,17,18,19]. Moreover, it has gained significant attention in the realm of beam propagation operation and control [20,21,22,23]. The concept of such lens was proposed by R. K. Luneburg in 1944 [6]. However, research progress was subjected to limitations of material science and processing capabilities. To validate this theory, a Luneburg lens comprising nested multilayered spherical shells with varying refractive indices was developed using polystyrene foaming in 1958 by G. D. M. Peeler [24]. Then, lots of efforts have been made to fabricate discrete spherical shells using quartz [25] and customized foams [26].

However, these approaches are limited by complex fabrication resources and high costs. To solve this issue, the theory of equivalent dielectric constant, where the spatial variation of the refractive index can be achieved by adjusting the geometrical parameters and air-dielectric ratio of the unit structures [27,28]. Approaches to alter the equivalent dielectric constant of the material include modifying thickness of the dielectric plate in the waveguide [29], creating holes of different shapes in the dielectric material [30], and also adjusting geometrical parameters of metamaterial unit cells [31,32]. Although these methods enhance fabrication accuracy and reduce costs, they are limited to producing two-dimensional (2D) lenses for generating plane waves. Although planar lenses have been widely used, they present certain disadvantages, such as beam scanning in only a single plane leading to an overall fan-shape beam [33], while volumetric lenses enable beam scanning in two planes, leading to much better coverage.

With the emergence of three-dimensional (3D) printing technology, all-dielectric-type Luneburg lenses have been considered for rapid and precise fabrication. Several designs have been proposed using non-enclosed unit cells, including equilateral triangles [34], mutually orthogonal cuboids with a cube at the center [35], cube with drilled holes [36], and lamellar structures [37]. Besides, two-dimensional Luneburg lenses have been designed as cylindrical [38,39,40] and plate lenses [41]. However, these unit cells show inherent anisotropy properties, which lead to sensitivity with regard to polarization changes. Hence, the elementary unit cell composing the 3D printed Luneburg lens needs to be spatially symmetric.

In light of the aforementioned issues, such as the exorbitant cost associated with traditional processing, limited single plane beam steering of 2D lenses and prevailing challenges posed by anisotropy and polarization sensitivity, this work proposes the design of a Luneburg lens based on isotropic unit cells and fabricated by 3D dielectric printing technology. The principal novelty of our lens in comparison to existing designs is the constituting metamaterial unit cell, which is constructed from a cubic all-dielectric material and an air sphere. Such unit cell is generally not proposed for additive manufacturing due to the complexity for 3D printing. However, in our work, we propose a technique to facilitate the 3D printing process, where the front face of a shell and the back face of the neighboring shell are printed together in a single step. In this way, we are able to achieve full spatial geometrical consistency of the lens, with a high degree of functional consistency transmitting electromagnetic waves in all directions. The design of the cells allows to modulate the effective permittivity from approximately 2 to 1, necessary to create the permittivity gradient of the lens. The lens can achieve high gain and low polarization sensitivity over a broad frequency range. Flexible antennas that can endure high curvature bending are designed at different frequencies to be exploited as primary feeds for the metamaterial-based lens. Measurements are carried out at multiple frequencies for the validation of the designed lens-antenna system and the experimental results are found to be in good agreement with the simulation ones. Finally, a beam scanning range covering up to ±72° is achieved, suggesting potential applications in fifth-generation (5G) and future sixth-generation (6G) wireless communication systems.

## 2. Design Principle

### 2.1. Configuration

**Figure 1:**
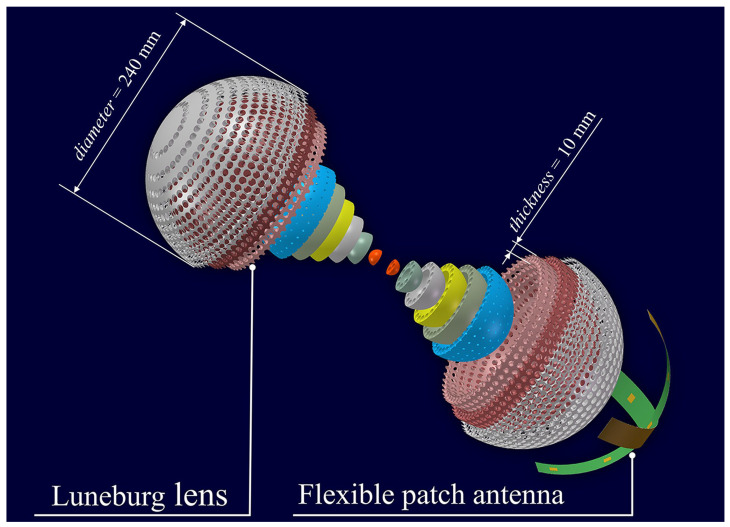
Schematic diagram of the lens-antenna system. The radiated spherical waves from the flexible patch antenna propagate through the Luneburg lens and are then transformed to planar waves. The Luneburg lens of diameter 240 mm is composed of 12 shells having each a thickness of 10 mm.

As shown in Figure 1, the proposed lens-antenna system comprises an all-dielectric Luneburg lens with a gradient effective permittivity and a flexible patch antenna array as feed. The lens is composed of 12 concentric dielectric spherical shells. Spherical waves emitted by the feed placed on the on the periphery pass through the Luneburg lens and are converted into highly directive plane waves, ultimately enabling the signal to be transmitted with high directivity, which is advantageous for long-distance communications and targeted coverage areas. According to the reciprocity theory, an incident collimated beam can be focused to a diagonally opposite point on the periphery of the lens, suggesting its further potential use as a receiver. The Luneburg lens belongs to the family of gradient-index lenses and the refractive index distribution is given as [42]:

(1) $$n\left(r\right)=\left\{\begin{array}{c} n_{0}\sqrt{2-{\left(\frac{r}{R}\right)}^{2},}\\ n_{0},\left(r>R\right) \end{array}\right.\left(0\leq r\leq R\right)$$

where R is the radius of the lens, r is the distance from the center of the lens and $n_{0}$ is the refractive index of the background medium (typically air) with a refractive index of 1.0. When the background medium is air, the relative relationship between the effective permittivity and refractive index is as follows:

(2) $$\varepsilon_{r}\left(r\right)={n\left(r\right)}^{2}=2-{\left(\frac{r}{R}\right)}^{2},\;\;\left(0\leq r\leq R\right)$$

where $\varepsilon_{r}\left(r\right)$ is the effective permittivity at the distance r from the center of the lens. From Equation (2), it is clear that the central region of the Luneburg lens has an effective permittivity of 2 and the effective permittivity gradually changes from the center toward the periphery of the sphere to reach a value of 1.

### 2.2. Design of the Luneburg Lens

The lens is designed based on the equal-thickness layering and equal effective permittivity variation layering methods. In this study, we adopt the relatively straightforward equal-thickness layering method proposed by Sanford [43]. A Luneburg lens with *N* shells and of radius R is considered such that the adjacent layers have a fixed thickness of 10 mm, and the effective permittivity of the ith layer $\varepsilon_{i}$ is determined at the center of its thickness, which can be mathematically expressed as [42]:

(3) $$r_{i+1}-r_{i}=\frac{R}{N}$$

(4) $$\varepsilon_{i}=\varepsilon_{r}\left(\frac{r_{i}+r_{i+1}}{2}\right)$$

The construction of the lens requires a gradient refractive index, which is achieved from the designed unit cell whose effective permittivity $\varepsilon_{eff}$ can be in a first step approximated by the Bruggeman’s model [44]:

(5) $$\frac{\varepsilon_{in}-\varepsilon_{eff}}{\varepsilon_{in}-\varepsilon_{ho}}=\left(1-p\right){\left(\frac{\varepsilon_{eff}}{\varepsilon_{ho}}\right)}^{\frac{1}{3}}$$

where $\varepsilon_{in}$ and $\varepsilon_{ho}$ are the permittivities of the inclusion (air) and host material (dielectric), respectively. p is the volume fraction of the inclusion material. Besides, the reflection and transmission coefficients obtained from full-wave simulations can also be exploited in the inversion method to accurately determine the effective permittivity of a unit cell [45].

### 2.3. Unit Cell Design

**Figure 2:**
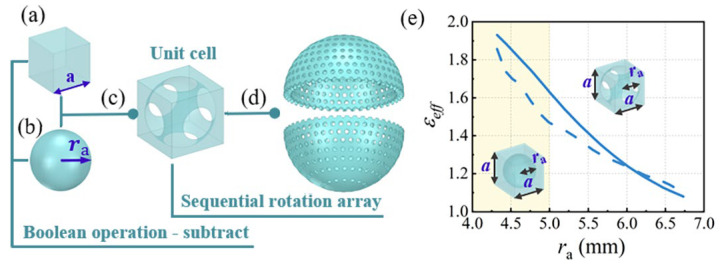
(**a**) All-dielectric cube with *a* = 10 mm. (**b**) Sphere of air of radius $r_{a}$. (**c**) Unit cell obtained from the dielectric cube and the air sphere. (**d**) One of the spherical shells composing the Luneburg lens. (**e**) Effective permittivity of the unit cells composed of an air sphere in the host dielectric material. A parametric analysis is performed to extract the effective permittivity value according to the radius $r_{a}$ of the air sphere in the dielectric cube. The yellow shaded zone represents the region where the value of radius $r_{a}$ is less or equal to *a*/2 ($r_{a}\leq a/2$). The continuous trace corresponds to approximate values calculated using the Bruggeman’s model and the dashed trace corresponds to accurate values retrieved from homogenization procedure at 6 GHz.

To achieve the effective permittivity of each layer, a metamaterial unit cell is designed. The elementary unit cell and one spherical shell of the Luneburg lens are depicted in Figure 2. By employing the Boolean operation, the structure of the unit cell can be constructed from a cubic all-dielectric material and an air sphere, as presented in Figure 2a–c. The size of the unit cell is set to 10 × 10 × 10 mm^3^, less than $\lambda_{0}/4$ at the frequency of 6 GHz. The radius of the air sphere is less than half the diagonal of the cube. A spherical shell, as depicted in Figure 2d, is then composed of lots of unit cells. This configuration, as a whole, is equivalent to perforation inside the spherical lens, exhibiting excellent physical-geometrical symmetry, and allows to achieve good electromagnetic and polarization characteristics.

As shown in Figure 2e, the relationship between the relative effective permittivity of the unit cell and radius of the air sphere is derived using the Bruggeman’s model (continuous trace) and inversion method (dashed trace). The FS3300PA material [46] with relative permittivity 2.54 and loss tangent 0.0012, is selected as the dielectric host material of the Luneburg lens. As the radius $r_{a}$ of the air sphere increases, the effective permittivity of the unit cell decreases, achieving a gradual change of the effective permittivity from 1.93 to 1.08.

Evidently, the greater the number of shells in the lens, the more effectively a smooth gradient of the effective permittivity from 2 to 1 can be achieved, thereby enhancing its performance. However, it has been shown that a saturation point exists in terms of the directivity when a certain number of layers is reached [47]. Moreover, increasing the number of layers increases the processing costs and poses challenges in terms of fabrication complexity, leading to larger processing errors. Therefore, to find a trade-off between fabrication complexity and optimal performance, assembly difficulty, and errors, the radius of 120 mm is adopted for the design of the lens. The entire structure consists of 12 shells with varying relative effective permittivity comprising a total of 3042 unit cells. The parameter $r_{a}$ of the air sphere with the corresponding effective permittivity of the unit cell and the number of unit cells in each spherical shell are listed in Table 1.

### 2.4. Design of the Feed Source

**Figure 3:**
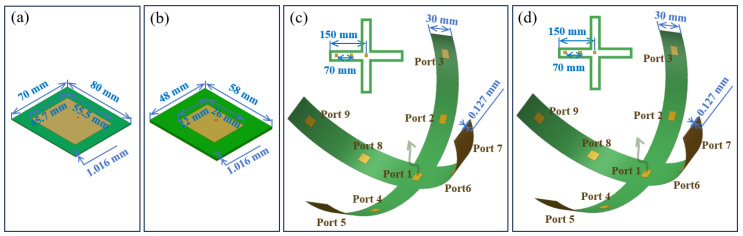
Schematic design of a patch antenna operating at (**a**) 2 GHz and (**b**) 4 GHz. The red dot shows the feeding point of the patch antennas. Design of the flexible multiport feed antennas operating at (**c**) 6 GHz, and (**d**) 8 GHz. The nine radiating elements are uniformly allocated with a spacing of 70 mm.

To illuminate the lens, an electromagnetic feeding source is required. A microstrip patch antenna is selected as the feed source based on its compact size, light weight, planar and conformal layout, excitation method and ease of integration. Since such type of antennas are known to exhibit narrowband frequency response, four different designs operating at 2 GHz, 4 GHz, 6 GHz and 8 GHz are respectively designed. For 2 GHz and 4 GHz, single patch antennas are designed, as shown in Figure 3a,b, while two flexible multiport antennas composed of 9 radiating elements are considered at 6 GHz and 8 GHz, as depicted in Figure 3c,d.

Since the dimensions of an elementary patch antenna at 2 GHz and 4 GHz are too large to form a flexible patch array with high performance, only a single patch radiator is used to feed the Luneburg lens at 2 GHz and 4 GHz. The size of the patch antenna is 45.7 mm × 55.5 mm and 22 mm × 26 mm, respectively, at 2 GHz and 4 GHz, as shown in Figure 3a,b. At 6 GHz and 8 GHz, the flexible multiport patch antenna has a cross-shaped centrosymmetric structure. For the frequency of 6 GHz, each radiating patch element has a size of 15 mm × 15 mm and the spacing between two patches is 70 mm. For 8 GHz, each radiating patch element has a size of 11.3 mm × 11.3 mm with a spacing between two patches of 70 mm. A F4B dielectric substrate of relative permittivity 2.65 and thickness 0.127 mm is used for the two flexible antennas. The thin substrate chosen for the high curvature bending enables the antenna to be tightly conformed to the spherical surface of the lens.

**Figure 4:**
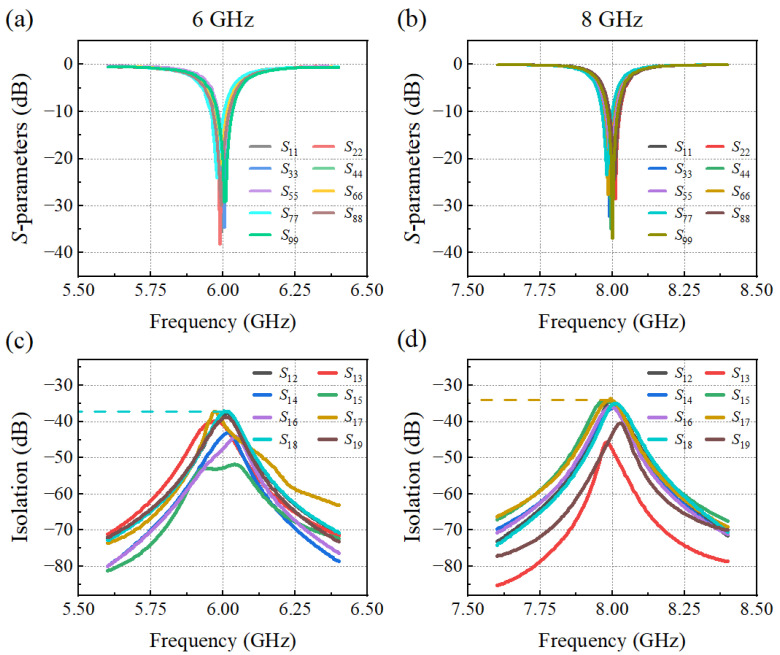
Matching responses (*S_xx_*-parameters) of the multiport patch antenna array operating at (**a**) 6 GHz, and (**b**) 8 GHz. Measured isolation between port 1 and the other ports at (**c**) 6 GHz, and (**d**) 8 GHz.

To demonstrate the characteristics of the flexible multiport antenna operating at 6 GHz and 8 GHz, a vector network analyzer (VNA) is employed for the measurement of the S-parameters. Figure 4a,b reveal a very slight frequency deviation for the different exciting ports, which primarily stems from the manufacturing and welding processes. In addition, the mutual influence of different radiation patches in one flexible antenna array is evaluated by the port isolation. The designed flexible antennas show good performances with an isolation of approximately 37 dB between two adjacent radiating elements at 6 GHz and 34 dB at 8 GHz, as shown in Figure 4c,d, which guarantees a good multi-beam generation to illuminate the Luneburg lens.

## 3. Fabrication and Measurement Results

**Figure 5:**
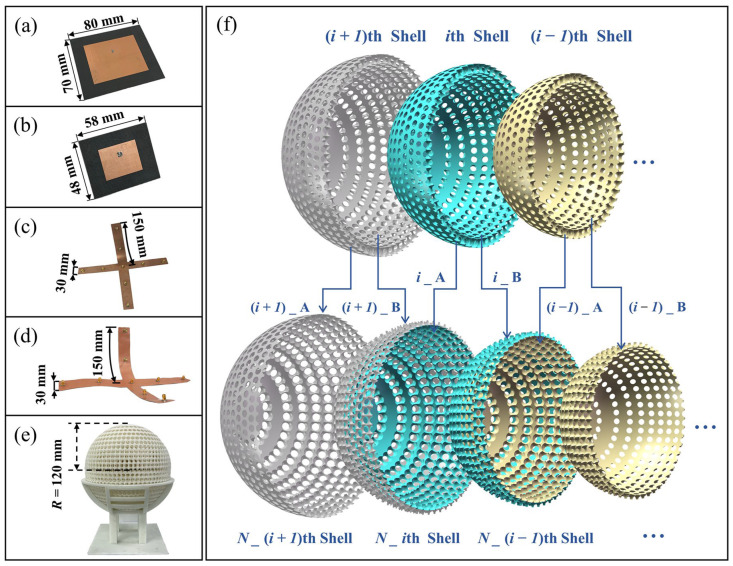
Photograph of the microstrip patch antenna operating at (**a**) 2 GHz and (**b**) 4 GHz. Photograph of the flexible multiport patch antenna operating at (**c**) 6 GHz, and (**d**) 8 GHz. (**e**) Photograph of the fabricated Luneburg lens. (**f**) Schematic diagram of the exploited printing method.

To validate the proposed design, the different feed antennas and the Luneburg lens are fabricated and the different photographs are depicted in Figure 5a–d. The antennas are manufactured by the common printed circuit board (PCB) processing technique, while the Luneburg lens is fabricated by 3D printing selective laser sintering (SLS) technique using eForm printing equipment [48], which can achieve a high fabrication accuracy.

It should be noted that the air sphere region needs to be supported by unsintered powder. When the radius of the air sphere $r_{a}$ is smaller than 5 mm, a closed spherical zone is formed inside the shell, and it is difficult to completely remove the powder inside the shell after printing. To solve this issue, an alternative printing method is proposed, as presented in Figure 5e, where the ith shell is splitted into the two parts i_A and i_B of equal thickness 5 mm. Then the part (i + 1)_B of the (i + 1)th shell and the part i_A of the ith shell are printed together as a new N_ith shell with the thickness of 10 mm. In this way, the closed spherical space of unit cell with $r_{a}$ less than 5 mm is opened such that the powder material can be easily removed. Each printed half shell is nested one into each other to form two parts of the lens and finally, the two half parts of the lens are fixed together to form the full lens by applying some glue at several contact points on the planar face of the outermost shell.

**Figure 6:**
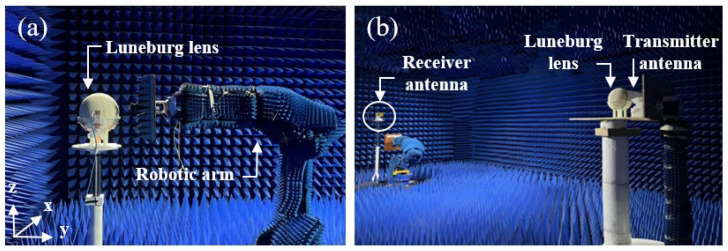
Photograph of the experimental setups. (**a**) Near-field measurement. (**b**) Far-field measurement.

Both the near-field and far-field experimental measurements of the Luneburg lens are performed in an anechoic chamber. For the near-field measurement, the setup is composed of the robotic arm with a field-sensing probe, as displayed in Figure 6a. The robotic arm can move in one plane with a step of 3 mm. Both the feed antenna and probe are connected to the vector network analyzer. For the far-field measurement setup, shown in Figure 6b, a horn antenna is used as receiver and is placed almost 6 m far from the Luneburg lens to collect the electric-field data in the far-field zone. The flexible antenna and the Luneburg lens are placed on a rotating plate and the far-field system is able to measure the radiation patterns for azimuthal angles in the ±90° range.

**Figure 7:**
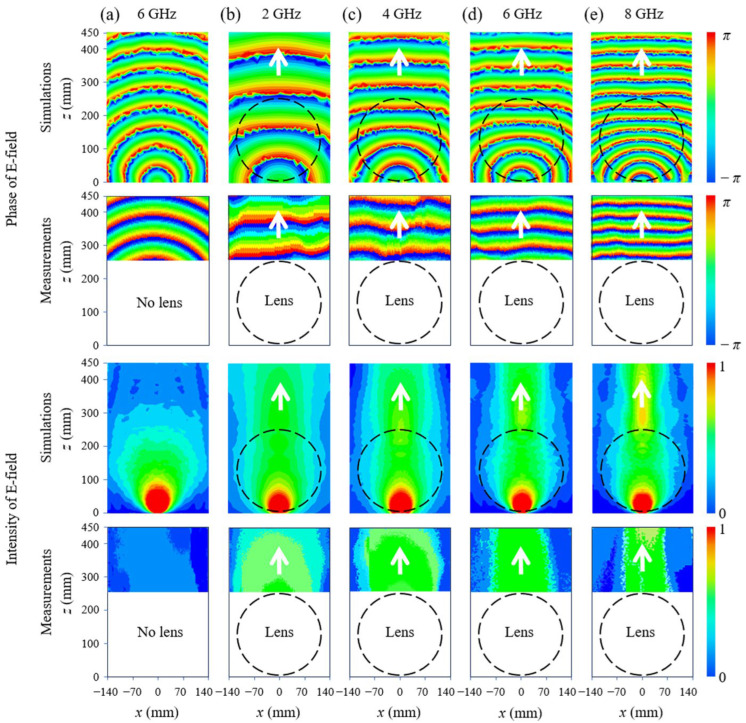
Near-field simulations and measurements. (**a**) Patch antenna alone at 6 GHz with only port 1 excited. Near-field results (intensity and phase) of the Luneburg lens-antenna with only port 1 excited at (**b**) 2 GHz, (**c**) 4 GHz, (**d**) 6 GHz and (**e**) 8 GHz.

To verify the broadband property of the 3D printed Luneburg lens-antenna, full-wave simulations and near-field measurements are performed from 2 GHz to 8 GHz. Both the intensity of the electric field and wavefront of the generated beam are displayed for the near-field results, as shown in Figure 7. The size of scanning area for the experimental measurements is 280 mm × 200 mm while the size of the simulated area is 280 mm × 450 mm. The near-field results of the patch antenna alone at 6 GHz used as a reference is presented in Figure 7a, where a spherical wave propagating along the *z*-axis with a curved wavefront can be clearly observed. The near-field results of the Luneburg lens-antenna are depicted in Figure 7b–e at 2 GHz, 4 GHz, 6 GHz, and 8 GHz, respectively, where the transformation from spherical waves to the plane waves is realized. A higher energy level of the plane wave can also be observed when compared to the spherical wave of the feed antenna at the same position. The good agreement between the simulations and measurements confirms the good implementation of the metamaterial unit cells to realize the required permittivity distribution of the Luneburg lens over a broad frequency range.

**Figure 8:**
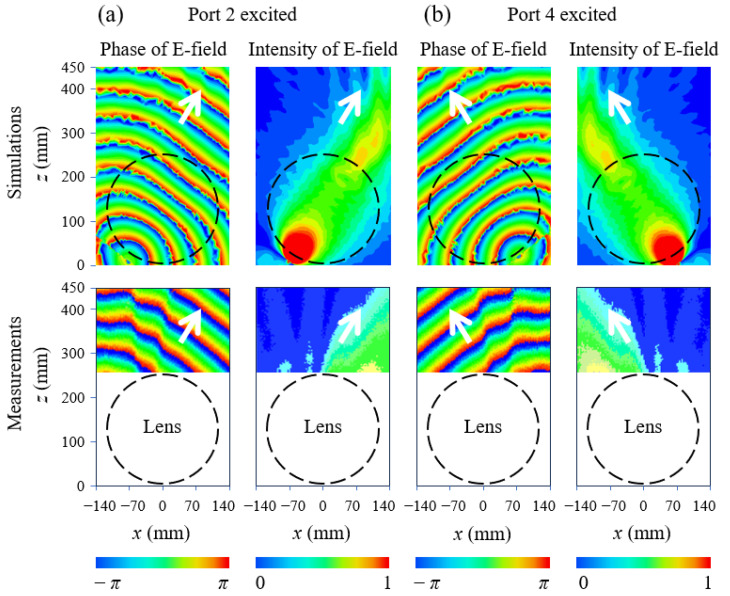
Near-field simulations and measurements of the Luneburg lens-antenna at 6 GHz. (**a**) Port 2 excited. (**b**) Port 4 excited.

The Luneburg lens has a good geometrical symmetry, such that it is able to support beam radiation in different directions, as validated at 6 GHz when port 2 and port 4 are respectively excited. A presented in Figure 8, the single beam appearing at ±36° is verified by exciting the ports 2 and 4 at the periphery of the Luneburg lens, which demonstrates that the spherical waves are converted to the plane waves at ±36°. Due to limitation in the movement of the robotic arm, near-field tests cannot be done to show the radiated beam when ports 3 and 5 are respectively excited. However, it should be noticed that the single beam appeared at other degrees can also be achieved by the designed Luneburg lens.

**Figure 9:**
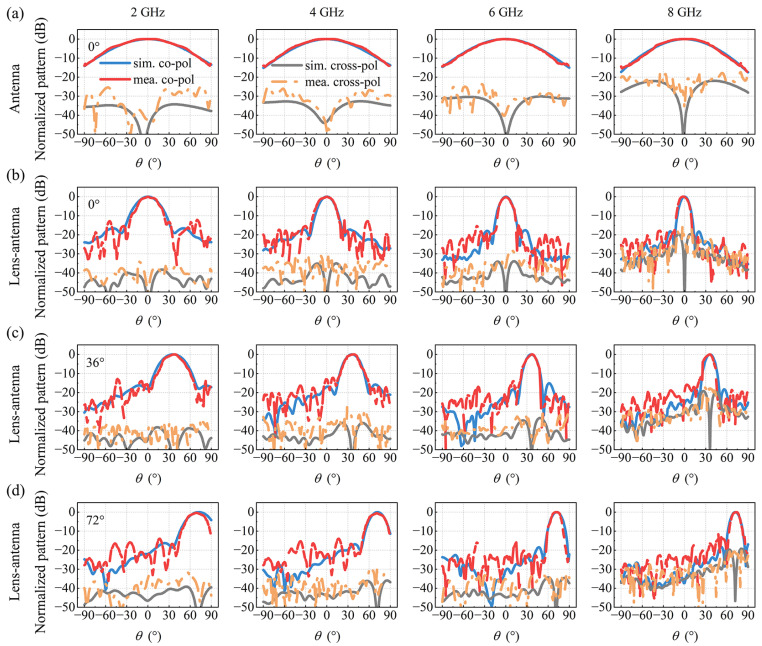
Simulated and measured far-field radiation patterns at 2 GHz, 4 GHz, 6 GHz and 8 GHz. (**a**) Feed antennas alone for boresight radiation. (**b**) Luneburg lens-antennas for boresight radiation. (**c**) Luneburg lens-antennas for 36° beam steering. (**d**) Luneburg lens-antennas for 72° beam steering.

Then, far-field simulations and measurements are carried out at 2 GHz, 4 GHz, 6 GHz and 8 GHz with port 1 excited for boresight radiation. The far-field patterns of the feed antenna alone are shown in Figure 9a, where a wide beam, typical characteristic of a microstrip patch antenna, is observed. Conversely, a narrower beam is obtained with the lens-antenna system, as shown in Figure 9b, indicating a good wavefront transformation of the designed Luneburg lens. The beam demonstrates a high degree of directivity, indicating the capacity of the lens-antenna to generate a directional and high-energy beam, which is favorable for long-distance transmissions. Moreover, as frequency increases, the directivity of the radiated beam increases due to the size of the lens, which becomes bigger when compared to wavelength, and the gain of the lens-antenna system is measured to be between 15.3 dBi to 22 dBi for the frequency varying from 2 GHz to 8 GHz. The half power beam width (HPBW) extracted from the experimental data shown in Figure 9b for boresight (0°) radiation is found to be 29.6° at 2 GHz, 19.2° at 4 GHz, 15.2° at 6 GHz, and 12° at 8 GHz. Meanwhile, when the beams are emitted at 36° and 72° separately, as depicted in Figure 9c,d, the same good beam convergence, highlighted by side lobes level lower than −10 dB, can be observed as for boresight (0°) radiation. Furthermore, the cross-polarization level is found to be lower than −15 dB in all cases, indicating a high polarization purity.

**Figure 10:**
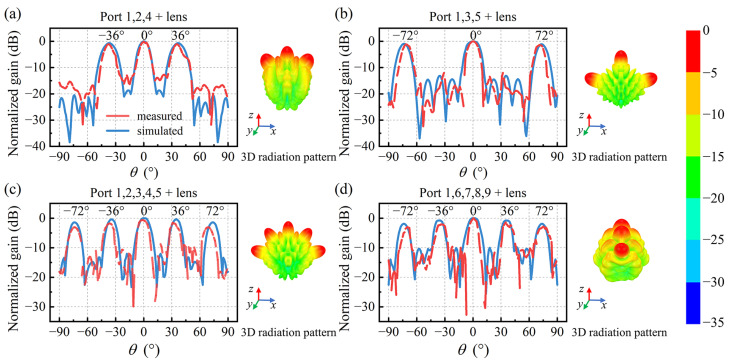
Simulated and measured far-field radiation patterns of the Luneburg lens-antenna at 6 GHz. (**a**) Ports 1, 2, and 4 excited simultaneously. (**b**) Ports 1, 3, and 5 excited simultaneously. (**c**) Ports 1, 2, 3, 4, and 5 excited simultaneously. (**d**) Ports 1, 6, 7, 8, and 9 excited simultaneously.

In order to validate the multi-beam feature of the Luneburg lens-antenna, far-field measurements of the Luneburg lens are performed at 6 GHz with multiple ports of antenna excited simultaneously. According to the geometric structure of the flexible antenna, the three beams are expected to be generated at 0° and ±36° when ports 1, 2, and 4 are simultaneously excited. When ports 1, 3, and 5 are simultaneously excited, the three beams are expected to be generated at 0° and ±72°. As it can be observed in Figure 10a,b, the simulated and measured radiation patterns are consistent with the expectations.

Additionally, the five beams configuration is also investigated at 6 GHz when the five ports located on the horizontal and vertical axes are respectively excited. The corresponding results are presented in Figure 10c,d, where the pointing angles can be observed at 0°, ±36° and ±72°. Compared to the beam at boresight (0°), the off-normal beams present a slightly lower gain due to inherent scanning losses. The slight discrepancies that are observed between simulations and measurements are attributed to fabrication tolerances and measurement errors arising from the power splitter and coaxial cable adapter. It is evident that the fabricated Luneburg lens-antenna can achieve multi-beam generation, together with a good port isolation. The scanning range of the lens-antenna can reach as wide as ±72° and the gain drop is less than 3 dB at maximum scan angle, which experimentally validates the wide scanning characteristics of the lens-antenna. When all five ports are simultaneously excited as shown by the results in Figure 10c, the scan losses are found to be around 1.8 dB at ±36° and 2.9 dB at ±72° from the experimental data.

Compared to other literature works summarized in Table 2, our proposed Luneburg lens-antenna can achieve broadband characteristics and wide scanning angle due to the judicious method of printing the different shells composed of air-dielectric unit cells. The low-loss FS3300PA dielectric material is used for its low relative permittivity such that it is relatively easy to realize an effective permittivity close to 1 using the proposed unit cell. Hence, our Luneburg lens can incorporate more layers under the processing accuracy limitations to realize a high gain antenna.

## 4. Conclusions

In summary, we have designed a unit cell that consists of a cubic material and an air sphere and proposed an alternative fabrication method for the manufacturing of a Luneburg lens using 3D printing technique. Owing to the isotropy exhibited by the unit cell and the omnidirectional uniformity achieved by the lens, low sensitivity to polarization is achieved. Three antennas, including an elementary radiating microstrip patch and two multiport patch antennas with low port-to-port isolation, are designed to illuminate the lens. Both numerical near-field and far-field simulations and experimental measurements are carried out in the frequency band ranging from 4 to 8 GHz. The properties of the Luneburg lens, including broadband, wide scanning angle and multiple beams generation, are validated. Three and five beams are realized using the Luneburg lens with the outmost beam located at approximately ±72°. The interference between beams is minimal, and it is straightforward to generate multiple beams in different directions. Furthermore, the peak gain of all beams is consistent, the coverage is uniform and the overall loss of the lens-antenna is low. When the aforementioned characteristics are considered in conjunction with the high gain, it is evident that the proposed Luneburg lens-antenna is capable of transmitting electromagnetic waves over long distances, showing potential applications for high-gain communications in 5G and future 6G wireless communication systems. Moreover, due to reciprocity, an incoming plane wave incident on the lens-antenna would be focused to a diagonally opposite point on the periphery of the lens, suggesting its further potential use as a receiver. A particular application scenario would be for direction of arrival (DOA) estimation due to its wide angular scanning ability [49].

## Figures and Tables

**Table 1:** Unit cell structural parameters for each shell.

**Shell**	**1**	**2**	**3**	**4**	**5**	**6**
$\varepsilon_{eff}$	1.93	1.88	1.84	1.73	1.63	1.54
$r_{a}$	4.32	4.42	4.56	4.79	5.00	5.22
Number of unit cells	3	16	38	71	113	164
**Shell**	**7**	**8**	**9**	**10**	**11**	**12**
$\varepsilon_{eff}$	1.42	1.33	1.24	1.18	1.13	1.08
$r_{a}$	5.46	5.73	6.05	6.24	6.46	6.73
Number of unit cells	227	299	381	473	573	684

**Table 2:** Comparison between the proposed Luneburg lens and relevant literature works.

Ref.	Center Frequency (GHz)	Material (*ε*_r_)	Radius (*λ*)	Max. Scanning angle (°)	Max. Gain (dBi)
[32]	10	Pla+ (2.54)	1.5	-	16
[34]	31.75	FLGPCL_02_ (2.9)	2.54	±50	19–21
[35]	5.8	SPR6000B (2.8)	~2.15	±30	13.5
[38]	10	Verowhiteplus (2.8)	~2.5	±29	19.8
[41]	33	Metal	2.75	±45	18.3
[44]	26.75	FLGPCL_02_ (2.9)	2.15	-	12
This work	6	3300PA (2.54)	2.4	±72	22

## Data Availability

Data are contained within the article.
